# Surgical Treatment of Lung Adenocarcinoma Tumor Thrombus Extending Into the Left Atrium: A Case Report

**DOI:** 10.1002/ccr3.72268

**Published:** 2026-03-12

**Authors:** Zhongjian Chong, Yu Tang, Aiping Zhang, Zijian Ma

**Affiliations:** ^1^ Department of Thoracic Surgery Nanjing First Hospital, Nanjing Medical University Nanjing China

**Keywords:** driver gene, left atrial tumor thrombus, local advanced stage, lung adenocarcinoma

## Abstract

When lung adenocarcinoma is accompanied by a tumor thrombus that extends through the pulmonary veins to the left atrium, it is an extremely rare case, and the disease is already at stage IIIA. The available treatment methods are also quite limited. This article reports a surgical treatment case of lung adenocarcinoma with a tumor thrombus extending to the left atrium. The patient underwent surgical treatment and has been followed up for three years with a good prognosis. This article aims to provide a reference for future clinical diagnosis and treatment of similar cases.

## 1 Introduction

Lung adenocarcinoma with a tumor thrombus extending into the left atrium via the pulmonary vein is an extremely rare clinical entity, and the optimal treatment is still controversial. The left atrial tumor thrombus may detach at any time, leading to the risk of death caused by mitral valve entrapment and systemic embolism. Although such patients often represent locally advanced disease, typically classified as stage IIIA, long‐term survival can be achieved if complete surgical resection, including left atrial tumor thrombectomy and radical lung cancer resection, is performed in combination with postoperative targeted therapy. Therefore, increasing awareness and understanding of this condition is of critical importance. This article reports a case of pulmonary adenocarcinoma presenting with a left atrial tumor thrombus. Comprehensive preoperative evaluation and preparation were conducted, and the patient received oral targeted therapy following surgery. The patient has been followed up for over two years with favorable recovery outcomes. Based on this case and a review of relevant literature from the past decade, a discussion on the clinical characteristics and management strategies is presented.

## 2 Case History

### 2.1 General Information

A 44‐year‐old female patient was admitted to the hospital due to “cough and expectoration for over 1 month, aggravated for 3 days.” The patient reported that over a month ago, without an obvious cause, she developed a cough with scanty, white, foamy sputum, which worsened when lying flat at night. She received intravenous antitussive medication for 3 days at a local hospital, but with poor therapeutic effect. Three days ago, her symptoms worsened. Chest CT revealed a mass in the right upper lobe of the lung, bilateral pleural effusion, and pericardial effusion. Pulmonary artery CTA showed a right upper lobe mass highly suggestive of lung cancer, left atrial myxoma, and thrombosis. The patient was previously in good health, had no history of tumor genetics, no history of smoking, and was a farmer by occupation. Physical examination on admission: Temperature 37.2°C, blood pressure 118/90 mmHg (1 mmHg = 0.133 kPa), heart rate 96 bpm, respiratory rate 20/min. The patient was alert but in poor general condition. Thorax symmetrical, bilateral lung movements normal; percussion revealed clear lung sounds; auscultation revealed harsh breath sounds bilaterally. No precordial bulge or thrill, cardiac borders are not enlarged, regular rhythm, no obvious pathological murmurs. Abdomen is soft and non‐tender, with no rebound tenderness, liver and spleen are not palpable below the costal margin. Negative shifting dullness is felt. Moderate pitting edema is present in both lower limbs. Biochemical indicators: NSE: 21.71 ng/mL; proBNP: 1355 ng/mL.

## 3 Imaging Data

### 3.1 Imaging Findings

**FIGURE 1:**
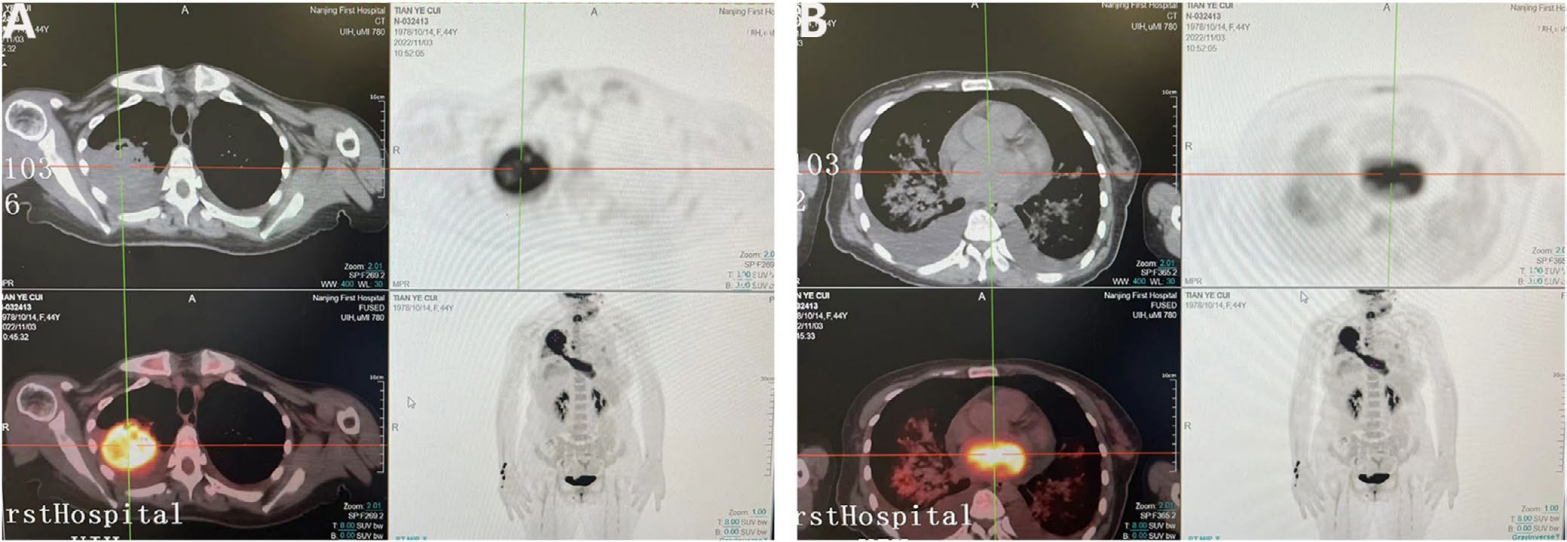
PET‐CT. (A) FDG‐avid mass in the right upper lobe measuring 72 mm × 68 mm, suggestive of a primary malignant lung tumor. (B) FDG‐avid mass in the left atrium extending along the right upper pulmonary vein and continuous with the pulmonary mass, suggestive of a tumor thrombus.

PET‐CT: Right upper lobe mass with increased FDG uptake (a mass in the right upper lung, with bronchial cutoff visible inside; maximum measured size ~72 mm × 68 mm on larger slice), accompanied by secondary distal localized atelectasis. Consider primary malignant lung tumor. Left atrial intraluminal mass‐like FDG hypermetabolic lesion extending along the right upper pulmonary vein, continuous with the intrapulmonary mass's FDG uptake—suggestive of tumor thrombus (Figure 1A,B). Mediastinal lymph nodes with increased FDG uptake (the 2nd and 4th groups of lymph nodes in the mediastinum), likely metastatic. Bilateral patchy high‐density shadows in the lungs, with only slightly increased FDG uptake—consider pulmonary edema. Imaging evaluation identified pericardial effusion, bilateral pleural effusions, and a small‐volume pelvic effusion. A cystic lesion with marginal FDG uptake was found in the right adnexal region, possibly a physiological follicle. Echocardiography revealed a left atrial mass with mitral valve diastolic obstruction. Consider high possibility of tumor metastasis or concomitant cancer‐related (or bland) thrombus formation (elliptical hyperechoic lesion measuring 83 mm × 31 mm in the left atrial cavity). Other etiologies not excluded; further clinical correlation and examination needed.

Left atrial enlargement; mild aortic regurgitation; mild mitral regurgitation; mild‐to‐moderate tricuspid regurgitation; moderate‐to‐severe pulmonary hypertension; normal left ventricular systolic function (Imaging was reviewed independently by radiologists and the cardiac team).

## 4 Differential Diagnosis, Investigations and Treatment

**FIGURE 2:**
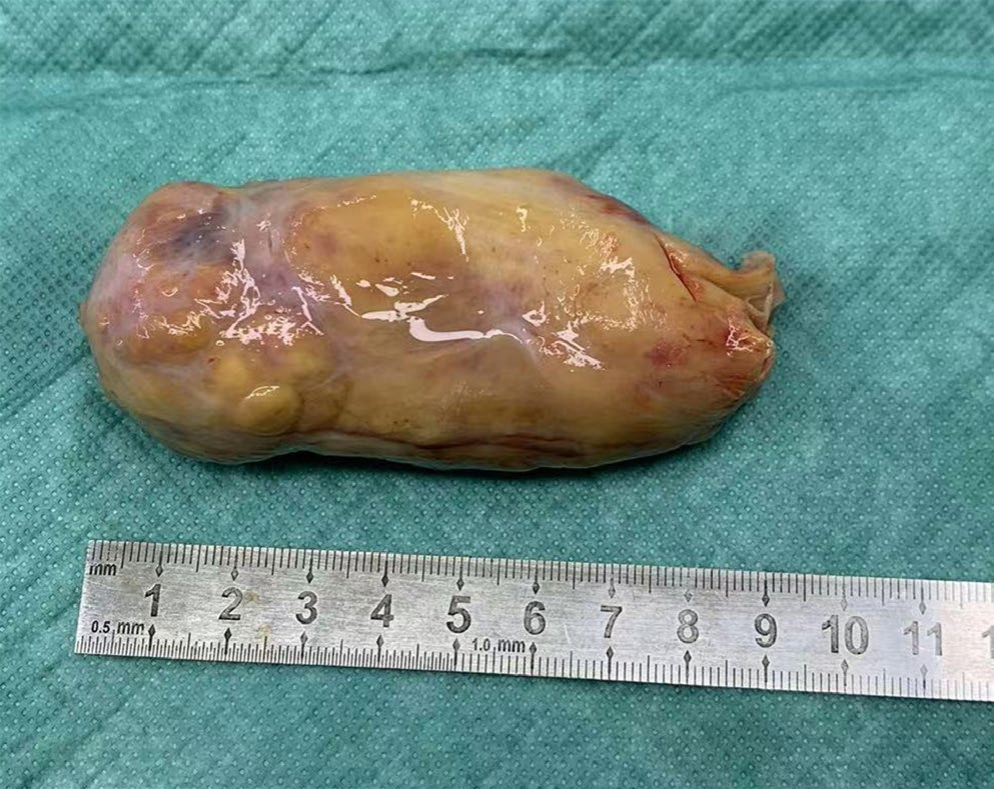
Gross specimen after resection of the left atrial mass (measuring 9 cm × 4 cm × 3 cm).

**FIGURE 3:**
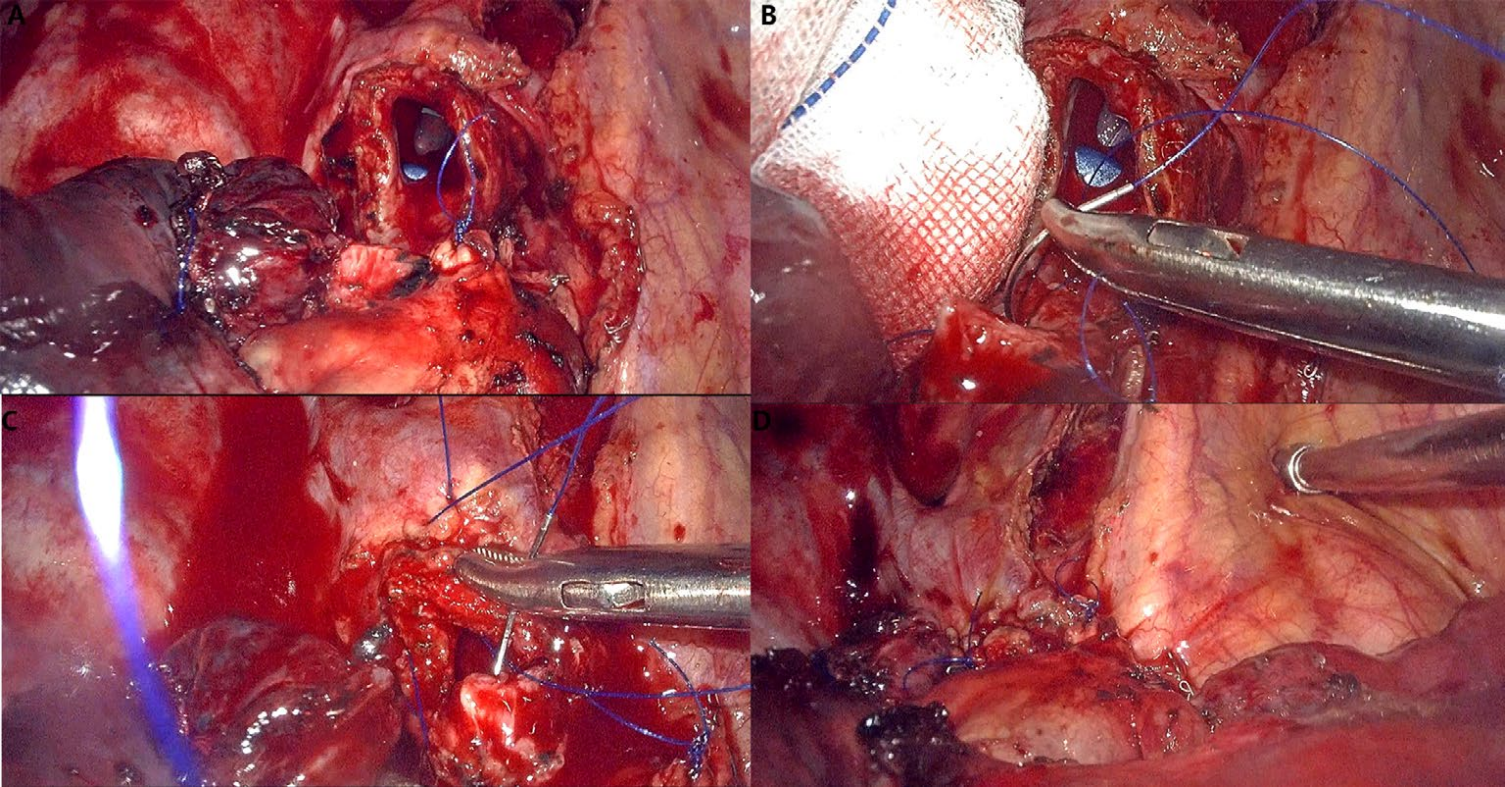
(A–D) Intraoperative schematic diagram (right upper lobe sleeve resection and systematic lymph node dissection).

The patient was admitted with acute heart failure. PET‐CT showed a left atrial mass‐like FDG hypermetabolic lesion extending along the right upper pulmonary vein, continuous with the intrapulmonary mass's FDG uptake—suggestive of a tumor thrombus. Diagnostic puncture of pericardial effusion and pleural effusion did not reveal any tumor cells. The tumor thrombus was large and could detach at any time, causing mitral valve entrapment and potentially leading to sudden cardiac death. Given the patient's frail condition, heart failure, moderate‐to‐severe pulmonary hypertension, large primary lung lesion, and pleural effusion, she first underwent left atrial mass resection under extracorporeal circulation (Figure 2). The postoperative course was stable. Postoperative pathology: Left atrium: epithelial‐derived malignant tumor, poorly differentiated, measuring 9 cm × 4 cm × 3 cm, with large areas of tumor necrosis. Based on immunohistochemistry and H&E staining, the findings were consistent with metastatic lung adenocarcinoma. Immunohistochemistry: ALK (+). Two weeks later, the patient underwent a sleeve resection of the right upper lobe of the lung and systematic lymph node dissection. (Figure 3A–D). The postoperative course was stable. Pathological findings: Right upper lung: malignant tumor with marked necrosis, suggestive of solid‐type invasive adenocarcinoma; tumor size 9 cm × 7 cm × 6 cm; tumor invasion of pleura; bronchial margin free of carcinoma. Lymph nodes:Group 2: metastasis in 1/3 nodes; Group 4: metastasis in 2/7 nodes; Group 7: 0/4 nodes negative for metastasis; Group 9: 0/2 nodes negative; Group 10: 0/2 nodes negative; Group 11: 0/4 nodes negative.

## 5 Conclusion and Results

The patient was admitted to the hospital presenting with acute heart failure symptoms and subsequently underwent left atrial tumor thrombectomy followed by radical resection of lung cancer. The total duration from postoperative recovery to discharge was 31 days. Following surgery, the patient received oral targeted drug therapy. Thirty months post‐operation, the patient has demonstrated favorable recovery with no signs of recurrence. Outpatient follow‐up examinations are scheduled every 6 months.

## 6 Discussion

Lung cancer metastasis refers to the process in which primary malignant tumors in the lung leave the original site and grow at distant locations through various routes. The most common metastatic sites are the lymph nodes, brain, bone, and liver [1, 2]. This process is extremely complex, often involving the lung cancer tumor microenvironment, lung cancer stem cells (LCSCs) [3, 4, 5], as well as mechanisms such as angiogenesis, epithelial–mesenchymal transition (EMT), lymphangiogenesis, and neovascularization [6, 7, 8].

Cardiac tumors are rare, with an incidence of approximately 0.001%–0.028% in clinical practice. Primary cardiac tumors are even rarer; most are metastatic tumors, among which lung cancer is the most common source. Lung cancer with cardiac metastasis is often underdiagnosed, with initial symptoms frequently presenting as cardiovascular manifestations. Clinical symptoms largely depend on the tumor's location, size, degree of invasion of surrounding structures, growth rate, and whether tumor fragments have detached. The main symptoms include heart failure, pericardial effusion, angina pectoris, and arrhythmia.

In the present case, the patient was a middle‐aged woman whose initial symptom was heart failure. Upon emergency admission, echocardiography and chest CT revealed a left atrial mass and a mass in the right upper lobe. PET‐CT further suggested a primary malignant lung tumor; the left atrial FDG‐avid mass extended along the right upper pulmonary vein and was continuous with the pulmonary mass, consistent with a tumor thrombus, without evidence of metastasis elsewhere. Literature review revealed that lung cancer with left atrial metastasis is rare, with few related reports both domestically and internationally. Most reported cases occur in patients with advanced disease and multiple metastases.

Lung cancer with isolated left atrial metastasis represents locally advanced disease, and the decision to proceed with surgery remains controversial. Radiotherapy or chemotherapy alone is generally ineffective, with a survival of only 3–6 months, and even with surgery, the median survival is only 11 months. The patient is young and exhibits no evidence of distant metastasis. However, a large tumor thrombus is present within the left atrium, posing a significant risk of embolization at any time, which could lead to mitral valve obstruction and potentially fatal outcomes. Given the patient's overall frailty, a concurrent median thoracotomy for left atrial mass resection and right upper lobectomy with sleeve resection was considered. However, this approach presents several challenges, including substantial surgical trauma, prolonged operative time, and the likelihood of incomplete lymph node dissection. Therefore, a staged surgical strategy was ultimately undertaken. After completing a preoperative evaluation, we first performed a left atrial mass resection under cardiopulmonary bypass. The operation and recovery were uneventful, and pathology confirmed metastatic lung adenocarcinoma (ALK positive).

After the first surgery, we discussed whether the patient should undergo curative lung resection, and whether neoadjuvant therapy was indicated. Considering her young age, the possibility of recurrent tumor thrombus extending into the left atrium, and her ALK‐positive status (enabling effective targeted therapy), we proceeded with the second surgery two weeks later. Preoperative echocardiography showed a smooth pulmonary vein lumen without a mass. The patient underwent a right upper lobectomy with sleeve resection and systematic lymph node dissection, with an uneventful postoperative course and discharge one week later. She was started on alectinib as adjuvant therapy. At our center's follow‐up for more than two years, she remains well without evidence of distant metastasis.

Mechanistic studies on cardiac metastasis from lung cancer are relatively scarce. In 2007, Sodha et al. were the first to identify the ALK rearrangement phenomenon in non‐small cell lung cancer (NSCLC) [9]. Under normal circumstances, the ALK protein activates intracellular signaling pathways by binding to extracellular ligands, promoting cell growth. When ALK fuses with echinoderm microtubule‐associated protein‐like 4 (EML4), multiple downstream pathways are activated, leading to the occurrence and progression of lung cancer, making ALK an important therapeutic target. Some studies have shown that ALK rearrangements are associated with increased rates of brain metastasis in NSCLC [10]. However, reports of cardiac metastases in ALK‐positive lung cancer are exceedingly rare, and genetic testing data are lacking; whether ALK mutations are associated with cardiac metastasis remains unclear. This patient's ALK‐positive result adds a valuable data point to this rare subset.

In conclusion, cardiac metastasis from lung cancer is rare and carries a poor prognosis, with limited research and few case reports. Clinicians should maintain a high index of suspicion for lung cancer in patients presenting with cardiovascular symptoms. For patients younger than 60 years without distant metastasis or extensive infiltration, surgery should be actively considered. The decision on simultaneous versus staged surgery should be individualized. Surgical resection combined with adjuvant therapy can effectively prolong survival in selected patients.

## Author Contributions

**Zhongjian Chong:** writing – original draft. **Yu Tang:** methodology. **Aiping Zhang:** investigation. **Zijian Ma:** conceptualization, writing – original draft.

## Funding

This work was supported by the National Natural Science Foundation of China Youth [Fund Number: 81902328/81702262].

## Consent

Written informed consent was obtained from the patient to publish this report in accordance with the journal's patient consent policy.

## Conflicts of Interest

The authors declare no conflicts of interest.

## Data Availability

The datasets used during the current study are available from the corresponding author on reasonable request.

## References

[1] T. Tamura , K. Kurishima , K. Nakazawa , et al., “Specific Organ Metastases and Survival in Metastatic Non‐Small‐Cell Lung Cancer,” Mol Clin Oncol 3, no. 1 (2015): 217–221. 25469298 10.3892/mco.2014.410 PMC4251107

[2] Q. Zhou , L. Zu , L. Li , et al., “Screening and Establishment of Human Lung Cancer Cell Lines With Organ‐Specific Metastasis Potential,” Zhongguo Fei Ai Za Zhi 17, no. 3 (2014): 175–182. 24667252 10.3779/j.issn.1009-3419.2014.03.20 PMC6019378

[3] W. S. Heng , R. Gosens , and F. A. E. Kruyt , “Lung Cancer Stem Cells: Origin, Features, Maintenance Mechanisms and Therapeutic Targeting,” Biochem Pharmacol 160 (2019): 121–133. 30557553 10.1016/j.bcp.2018.12.010

[4] D. Pankova , Y. Jiang , M. Chatzifrangkeskou , et al., “RASSF1A Controls Tissue Stiffness and Cancer Stem‐Like Cells in Lung Adenocarcinoma,” EMBO J 38, no. 13 (2019): e100532. 31268606 10.15252/embj.2018100532 PMC6600643

[5] M. M. S. Balla , H. D. Yadav , and B. N. Pandey , “Tumorsphere Assay Provides a Better In Vitro Method for Cancer Stem‐Like Cells Enrichment in A549 Lung Adenocarcinoma Cells,” Tissue Cell 60 (2019): 21–24. 31582014 10.1016/j.tice.2019.07.003

[6] M. Yang , E. Arai , Y. Takahashi , et al., “Cooperative Participation of Epigenomic and Genomic Alterations in the Clinicopathological Diversity Of Gastric Adenocarcinomas: Significance of Cell Adhesion and Epithelial‐Mesenchymal Transition‐Related Signaling Pathways,” Carcinogenesis 41, no. 11 (2020): 1473–1484. 32710740 10.1093/carcin/bgaa079 PMC7665242

[7] H. Canever , P. S. Carollo , R. Fleurisson , P. P. Girard , and N. Borghi , “Molecular Tension Microscopy of E‐Cadherin During Epithelial‐Mesenchymal Transition,” Methods Mol Biol 2179 (2021): 289–299. 32939728 10.1007/978-1-0716-0779-4_22

[8] D. Cao , G.‐Y. Zhu , Y. Lu , et al., “Luteolin Suppresses Epithelial‐Mesenchymal Transition and Migration of Triple‐Negative Breast Cancer Cells by Inhibiting Yap/Taz Activity,” Biomed Pharmacother 129 (2020): 110–462. 10.1016/j.biopha.2020.110462 32768952

[9] M. Soda , Y. L. Choi , M. Enomoto , et al., “Identification of the Transforming EML4‐ALK Fusion Gene in Non‐Small‐Cell Lung Cancer,” Nature 448, no. 7153 (2007): 561–566. 17625570 10.1038/nature05945

[10] M. Preusser , A. S. Berghoff , A. Ilhan‐Mutlu , et al., “ALK Gene Translocations and Amplifications in Brain Metastases of Non‐Small Cell Lung Cancer,” Lung Cancer 80, no. 3 (2013): 278–283. 23453647 10.1016/j.lungcan.2013.01.019

